# A Cobalt‐Based Metal‐Organic Framework Nanosheet as the Electrode for High‐Performance Asymmetric Supercapacitor

**DOI:** 10.1002/advs.202207545

**Published:** 2023-04-23

**Authors:** Qian Liu, Zengqi Guo, Cong Wang, Su Guo, Zhiwei Xu, Chenguang Hu, Yujing Liu, Yalei Wang, Jun He, Wai‐Yeung Wong

**Affiliations:** ^1^ Anhui Province Key Laboratory of Functional Coordinated Complexes for Materials Chemistry and Application School of Chemical and Environmental Engineering Anhui Polytechnic University Wuhu 241000 P. R. China; ^2^ Department of Applied Biology and Chemical Technology and Research Institute for Smart Energy The Hong Kong Polytechnic University Hung Hom, Kowloon Hong Kong P. R. China; ^3^ School of Chemical Engineering and Light Industry Guangdong University of Technology Guangzhou 510006 P.R. China

**Keywords:** 2D material, bottom‐up method, electrochemistry, MOF nanosheet, supercapacitor

## Abstract

Inspired by the significant advantages of the bottom‐up synthesis whose structures and functionalities can be customized by the selection of molecular components, a 2D metal‐organic framework (MOF) nanosheet Co‐BTB‐LB has been synthesized by a liquid–liquid interface‐assisted method. The as‐prepared Co‐BTB‐LB is identified by scanning electron microscopy/energy dispersive spectroscopy (SEM/EDX) and X‐ray photoelectron spectroscopy (XPS), and the sheet‐like structure is verified by scanning electron microscopy (SEM), high‐resolution transmission electron microscopy (HRTEM), and atomic force microscopy (AFM). Co‐BTB‐LB electrode exhibits an excellent capacity of 4969.3 F g^−1^ at 1 A g^−1^ and good cycling stability with 75% capacity retention after 1000 cycles. The asymmetric supercapacitor device with Co‐BTB‐LB as the positive electrode shows a maximum energy density of 150.2 Wh kg^−1^ at a power density of 1619.2 W kg^−1^ and good cycling stability with a capacitance retention of 97.1% after 10000 cycles. This represents a state‐of‐the‐art performance reported for asymmetric supercapacitor device using electroactive bottom‐up metal‐complex nanosheet, which will clearly lead to a significant expansion of the applicability of this type of 2D nanomaterials.

## Graphical abstract

Bottom‐up 2D metal‐complex nanosheet Co‐BTB‐LB exhibits good electrochemical properties and cycling stability, and the ASC device with Co‐BTB‐LB as the positive electrode also exhibits high energy density and good cycling stability, which represents a state‐of‐the‐art supercapacitor performance for electroactive bottom‐up nanosheet reported.

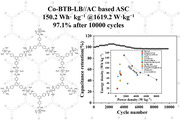

## 1 Introduction

With the increase in awareness of environmental protection, topics on renewable energy sources and sustainable energy storage devices are getting more important in the scientific community.^[^ ^1^ ^]^ Sutherland pointed out that the economical long‐term energy storage for stationary applications is a pivotal missing element toward enabling a predominantly renewable energy powered future society.^[^ ^2^ ^]^ Among the most common energy storage devices (e.g., supercapacitors, batteries, fuel cells, and electrochromic devices), research on supercapacitors has attracted an increasing attention in recent years because of their high capacitance, good stability, fast charge/discharge processes, and low maintenance cost.^[^ ^3^ ^]^ However, their narrow voltage window and low energy density severely hinder further application.^[^ ^4^ ^]^ The charge storage capability of supercapacitors mainly depends on the electrode materials, which need to be further developed.^[^ ^5^ ^]^

Since the discovery of graphene,^[^ ^6^ ^]^ nanosheets with 2D polymeric structures, such as silicone,^[^ ^7^ ^]^ germanene,^[^ ^8^ ^]^ stanene,^[^ ^9^ ^]^ and phosphorene,^[^ ^10^ ^]^ have attracted much attention in the fields of electronics, batteries and supercapacitors, which promote the research and development of other 2D nanosheet materials. Park and co‐workers addressed the emerging nanotechnologies that enable extrinsic pseudocapacitance in 2D nanomaterials, the performances of which can be significantly improved by controlling the structure at the microscopic level such as crystalline phase, defects, pores, and size to the macroscopic level such as morphology and hierarchical structure.^[^ ^11^ ^]^ Inspired by the significant advantages of unique electronic structures and physical properties, 2D nanosheets are very attractive candidates for supercapacitors in the pursuit of achieving good electrochemical performance because their ideal ordered nanopores and interlayer space for ion intercalation/deintercalation are beneficial for preserving the ion diffusion channel and accelerating the electron transfer. Therefore, the construction of new 2D materials for energy storage and conversion becomes one of the key critical strategies for their future applications.^[^ ^12^ ^]^ For example, Park and co‐workers showed that by coupling the nanosheets of oxidized black phosphorus (oBP) and reduced graphene oxide (rGO), the surface redox pseudocapacitance of 478 F g^−1^ (99% of total stored charge) was achieved, which exceeds the kinetic and stability limitations of previously explored BP.^[^ ^13^ ^]^ The representative 2D nanosheets mainly include surface 2D metal‐organic frameworks (MOFs)^[^ ^14^ ^]^ and covalent organic frameworks (COFs),^[^ ^15^ ^]^ in which 2D MOFs with high specific surface areas are good electrode materials for supercapacitors due to their adjustable morphology, designable porous structure and interlayer spacing.^[^ ^16^ ^]^ Hydrothermal method and top‐down exfoliation are the typical strategies to obtain 2D MOF materials. For example, Chen and co‐workers have synthesized 2D hierarchical porous cobalt/nickel‐based vanadates thin sheets via a hydrothermal method by direct decomposition of the mixed aqueous solution of NiCl_2_/CoCl_2_ and NaVO_3_, and the sheet‐based electrode showed a remarkable specific capacity of 848.5 C g^−1^ (specific capacitance of 2617.5 F g^−1^) at 1 A g^−1^.^[^ ^17^ ^]^ Xia and co‐workers prepared the Co‐Ni‐B‐S MOFs by a hydrothermal method followed by the boronization and subsequent sulfurization, which exhibited a high specific capacitance of 1281 F g^−1^ at 1 A g^−1^, and outstanding cycling stability of 92.1% retention after 10 000 cycles.^[^ ^18^ ^]^ Dai and co‐workers fabricated a nanosheet MoS_2_@carbon, which delivered a specific capacitance of 1302 F g^−1^ at a current density of 1.0 A g^−1^ and showed a 90% capacitance retention after 10 000 charging–discharging cycles.^[^ ^19^ ^]^ Fu and co‐workers synthesized a 2D Co‐catecholate (Co‐CAT) which delivered an exceptionally high capacity of 1160 F g^−1^ at 1 A g^−1^ and a special self‐discharge rate (86.8% after 48 h).^[^ ^20^ ^]^ Huang and co‐workers fabricated 2D mesh‐like vertical structures (NiCo_2_S_4_@Ni(OH)_2_) by hydrothermal method, and the resultant flexible electrode exhibited a high areal capacity of 535.9 µAh cm^−2^ (246.9 mAh g^−1^) at 3 mA cm^−2^ and outstanding rate performance with 84.7% retention at 30 mA cm^−2^.^[^ ^21^ ^]^ Hao and co‐workers fabricated ultrafine Ni(OH)_2_ nanosheets grown on 3D graphene hydrogel by electrochemical exfoliation for supercapacitor applications, which showed a specific capacity of 1603 F g^−1^.^[^ ^22^ ^]^ Although the hydrothermal method is an effective way to afford nanosheet materials, this strategy usually requires high temperature, which often leads to uncontrollable growth of nanomaterials with serious agglomeration that has hindered its large‐scale application in supercapacitors. It has been reported that the properties of top‐down nanosheets prepared by exfoliation of bulk‐layered crystalline mother materials are greatly limited by their mother materials.^[^ ^23^ ^]^ Recently, MOF nanosheets were produced by liquid–liquid (L–L) interfacial synthesis with an aqueous solution of metal ions and an organic solution of ligands.^[^ ^24^ ^]^ Compared with the hydrothermal method and top‐down exfoliation strategies, L–L interfacial method is a controllable synthesis. A significant advantage of the bottom‐up synthesis of nanosheet is that the structures can be customized through the choice of components (ligands and metal ions). More importantly, the redox‐active organic linkers and variable valence of metal centers enable MOF nanosheets with graphene‐like structures to possess high porosity, high surface area, and a lot of active sites. Very recently, the metal‐carboxylate coordination bond is one of the most important chemical bonds in MOFs reported.^[^ ^25^ ^]^ The balance between metal‐carboxylate bond strength and its reversibility is useful in forming superstructures with ordered nanopores and layered structures. For example, Kern and co‐workers first used the metal‐carboxylate coordination bond to create a 2D polymer structure at a well‐defined metal surface under ultrahigh vacuum (UHV) conditions.^[^ ^26^ ^]^ Nevertheless, while pyrrole and pyridine derivatives as excellent N‐donor ligands have been used to construct numerous 2D nanosheets, reports on 2D nanosheets constructed from carboxylate‐based ligands are still very limited.

Given this background, we have been working on the synthesis of the optoelectronic bottom‐up nanosheets,^[^ ^27^ ^]^ and found that there are scarce reports on the application of bottom‐up metal‐complex nanosheets for supercapacitors. Herein, we report a stable and easily prepared MOF nanosheet Co‐BTB‐LB synthesized from 1,3,5‐tris(4‐carboxyphenyl)benzene (H_3_BTB) ligand and cobalt(II) nitrate hexahydrate. In view of the fact that superstructure with ordered nanopores and layered structure can provide much more active sites for the as‐synthesized nanosheet Co‐BTB‐LB, it is expected to be readily applied in asymmetric supercapacitor (ASC). As expected, Co‐BTB‐LB exhibits good electrochemical properties of high capacity of 4969.3 F g^−1^ and discharge time of 2236 s at the current density of 1 A g^−1^ and good cycling stability with 75% capacity retention after 1000 cycles. Furthermore, the ASC device with Co‐BTB‐LB as the positive electrode shows a maximum energy density of 150.2 Wh kg^−1^ at a power density of 1619.2 W kg^−1^ and good cycling stability with a capacitance retention of 97.1% after 10 000 cycles performed at a current density of 10 A g^−1^, which reveal the potential applications of nanosheet Co‐BTB‐LB in energy‐storage devices.

## 2 Results and Discussion

### 2.1 Materials Preparation and Characterization

#### 2.1.1 Preparation of Nanosheet Co‐BTB‐LB by Bottom‐Up Method

Degassed CH_2_Cl_2_ solution of the ligand H_3_BTB (0.75×10^−4^ mol L^−1^, 10 mL) was added into a glass vial with a volume of 50 mL and a diameter of 3.2 cm. Solutions were then covered with degassed D.I. water (10 mL) to form a buffer layer before the addition of 10 mL water solution of Co(NO_3_)_2_·6H_2_O (5.0×10^−2^ mol L^−1^). The reaction was allowed to proceed for 15 days at room temperature. After that, nanosheet emerged at the interface as a layered film, which was marked as Co‐BTB‐LB. After the removal of aqueous and organic phases, the as‐prepared nanosheet was washed thoroughly with water, ethanol, and CH_2_Cl_2_, and dried in vacuo.

#### 2.1.2 Preparation of the Bulk Co‐BTB‐HT by Hydrothermal Method

1.16 g of Co(NO_3_)_2_·6H_2_O was added to a degassed DMF solution of the ligand H_3_BTB (0.75×10^−4^ mol L^−1^, 80 mL), and the mixture was sonicated for 10 min in an ultrasound system. Then, the mixed solution was shifted into a Teflon‐lined stainless steel autoclave (100 mL capacity) and heated at 170 °C for 12 h. After the mixture was cooled to room temperature naturally, the obtained brown precipitate was thoroughly washed and centrifuged with DMF several times. Finally, the collected sample was placed in a vacuum oven at 60 °C and dried for 24 h. The as‐prepared sample was marked as Co‐BTB‐HT.

#### 2.1.3 Structure of Nanosheet Co‐BTB‐LB

**Figure 1:**
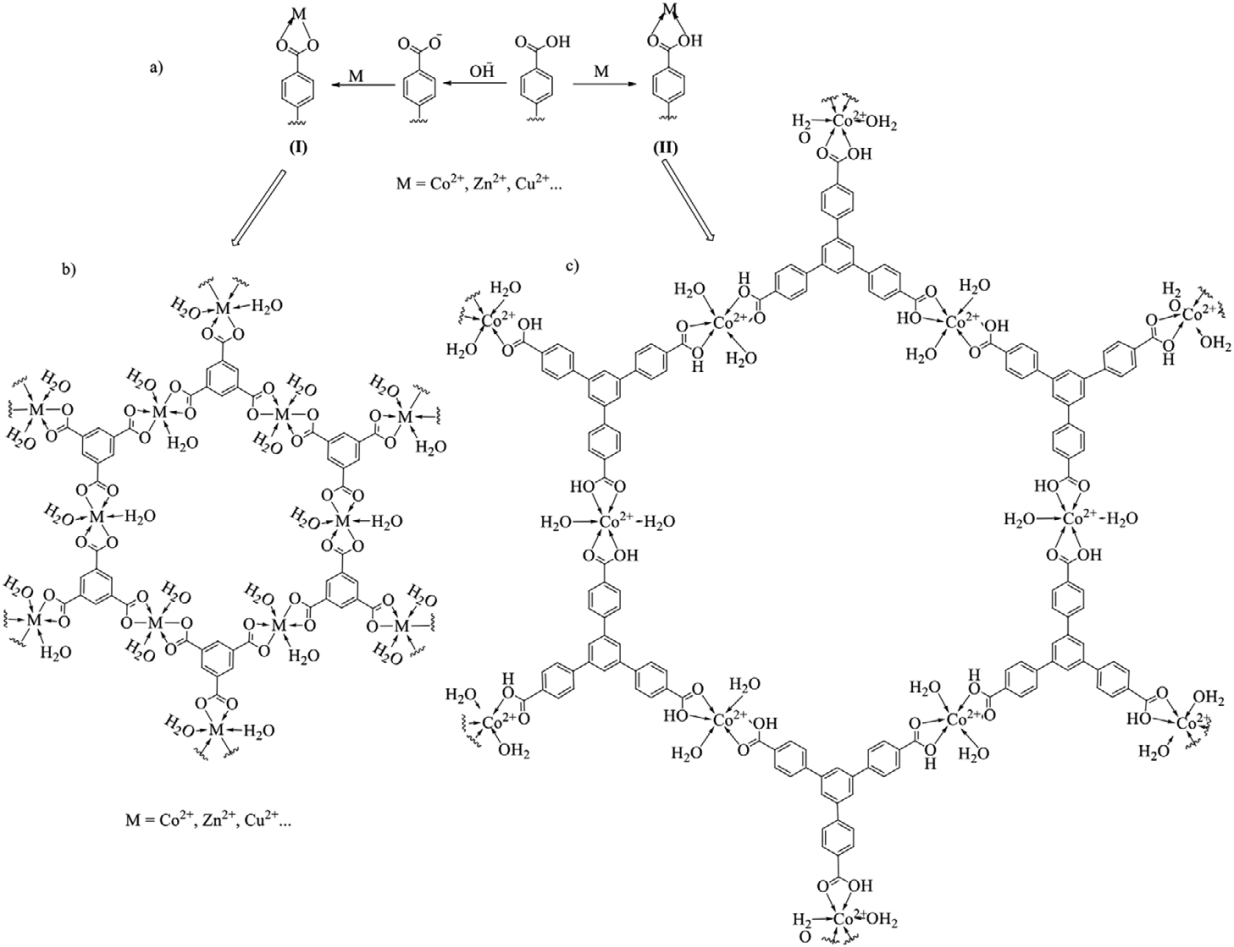
a) Different types of interactions of M (Co^2+^, Zn^2+^, Cu^2+^…) with the ligand unit containing carboxyl group.^[^ ^28^ ^]^ b) Structure of the unit for H_3_BTB‐based MOFs (pH of the reaction solution was adjusted to 7 by using a NaOH solution).^[^ ^29^ ^]^ c) Structure of the repeat unit for Co‐BTB‐LB with NO_3_^−^ omitted for clarity (this work).

**Figure 2:**
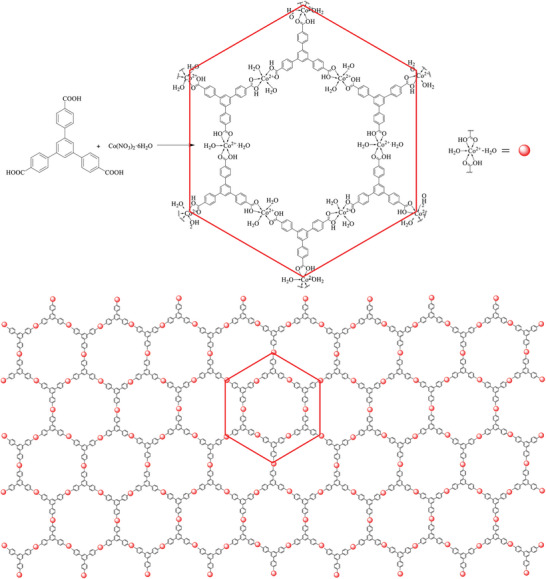
Schematic illustration and topological structure of the nanosheet Co‐BTB‐LB derived from Co^2+^ ion and H_3_BTB (ligand).

The MOF nanosheet Co‐BTB‐LB has been synthesized between the metal ion Co^2+^ and ligand H_3_BTB with the topological structure depicted in Figure 2. According to the literature reports,^[^ ^28^ ^]^ there are different types of interactions of M (M = Co^2+^, Zn^2+^, Cu^2+^…) with the ligand unit containing carboxyl group. As shown in **Figure** **1**a, when the pH of the reaction solution was adjusted to 7 by using NaOH solution, the type of interaction between M^2+^ and carboxyl group is represented in (I), which was also confirmed by the structures of MOFs in the literature (Figure 1b).^[^ ^29^ ^]^ When the pH of the reaction solution is not adjusted, the type of interaction is indicated in (II). In order to confirm the type of interaction of Co‐BTB‐LB, a small molecular model M1 has been synthesized as depicted in Scheme S1 (Supporting Information). 3,5‐Diisopropylbenzoic acid was selected as the ligand to react with Co^2+^ in D.I. water without pH adjustment at room temperature, and the reaction condition of which was the same as that in the synthesis of Co‐BTB‐LB. The product M1 has been characterized by proton nuclear magnetic resonance (^1^H NMR), fourier‐transform infrared spectoscopy (FT‐IR) and liquid chromatography‐mass spectrometry (LC‐MS). As shown in Figure S2 (Supporting Information), the proton signal of carboxyl group for M1 still exists, which is shifted by 0.05 ppm in the downfield region (*δ*, above 12.84 ppm) compared with that for the ligand 3,5‐diisopropylbenzoic acid as depicted in Figure S3 (Supporting Information), and the proton signals of aromatic rings and CH_3_ also have different degrees of shifting between them, indicating that the type of interaction of M1 is represented as (II). LC‐MS was employed to confirm the product by the respective molecular ion peak and fragment as shown in Figure S4 (Supporting Information). The FT‐IR spectra of M1 and nanosheet Co‐BTB‐LB are given in Figure S5 (Supporting Information) and the data are summarized in Table S1 (Supporting Information). Compared with the ligand, the Ar‐COOH, C=O, C=C and C‐O vibrational bands also exist, while only the shape and intensity of the peaks have been changed. The coordination between Co^2+^ and H_2_O molecules resulted in the appearance of strong vibrational bands in the spectral range of 3429 and 798–783 cm^−1^ for M1 and nanosheet Co‐BTB‐LB. Upon the coordination of H_3_BTB with Co^2+^, the position of C=O is shifted to lower wavenumbers for M1 and nanosheet Co‐BTB‐LB, which indicates that the carboxylic oxygen is coordinated by Co^2+^. In addition, the appearance of Co‐O vibrational bands of M1 and nanosheet Co‐BTB‐LB at 596 and 582 cm^−1^ also confirms the coordination behavior of H_3_BTB and Co^2+^.^[^ ^29^ ^]^ Besides, it is stable in air and in 1 M KOH aqueous solution (Figure S5 and Table S1, Supporting Information). To sum up, the topological structure of nanosheet Co‐BTB‐LB has been simulated and depicted in Figures 1c and **2**. Here, NO_3_^−^ from Co(NO_3_)_2_·6H_2_O functions as a counter anion for such cobalt complex motif, and the topological structures of the nanosheet Co‐BTB‐LB is illustrated accordingly (NO_3_^−^ is omitted for clarity).

#### 2.1.4 Morphology of Nanosheet Co‐BTB‐LB

**Figure 3:**
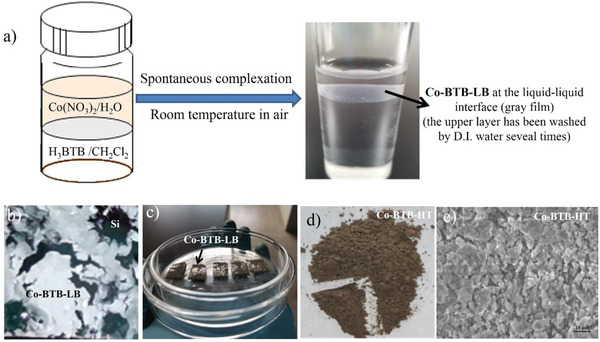
a) Schematic illustration and photographs of the liquid‐liquid interfacial synthesis. MOF nanosheet Co‐BTB‐LB transferred onto b) Si and c) Ni foam substrates. d) Photograph of the Co‐BTB‐HT from the reaction by hydrothermal method. e) SEM image of Co‐BTB‐HT on a Si substrate.

**Figure 4:**
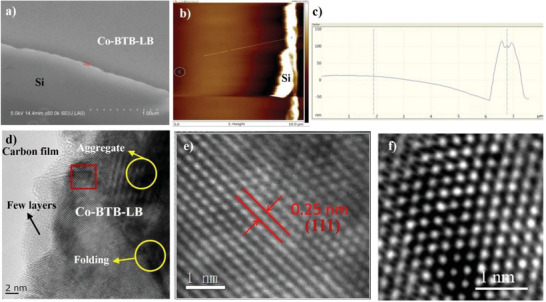
a) SEM image for Co‐BTB‐LB on a Si substrate. Scale bar, 1 µm. b) AFM image for Co‐BTB‐LB on Si substrate. Scale bar, 10 µm. c) Cross‐section analysis at one of the steps in the scratched region in (b). d) HRTEM image for Co‐BTB‐LB on ultrathin pure carbon film with no formvar backing on lacey carbon support film. Scale bar, 2 nm. e) The lattice distance of hexagonal structure shown in the white square in (d). Scale bar, 1 nm. f) Close‐up of the hexagonal pattern shown in the white square in (d). Scale bar, 1nm.

As shown in **Figure** **3**a, at room temperature, a spontaneous coordination reaction led to the generation of nanosheet Co‐BTB‐LB at the water/oil interface, which appeared as a gray film. Nanosheet Co‐BTB‐LB can be transferred from the interface onto various substrates, such as on silicon (Si) or nickel (Ni) foam substrates (Figure 3b,c). A conventional hydrothermal method was also performed in *N,N*‐dimethylformamide at 170 °C, resulting in a solid material (Co‐BTB‐HT) far from a film texture (Figure 3d). Its disordered structure was verified by scanning electron microscopy (SEM) in Figure 3e, which shows an irregular block lacking a uniform and continuous structure compared with nanosheet Co‐BTB‐LB. Also, X‐ray photoelectron spectroscopy (XPS) has been conducted to verify its disordered structure (vide infra). Co‐BTB‐LB is not soluble in either water or organic solvent, reflecting the polymeric structure as proposed in Figure 2. The Co^2+^ ion is six‐coordinated in a distorted octahedral coordination geometry, completed by four carboxylic oxygen atoms from two H_3_BTB ligands and two oxygen atoms from two H_2_O, forming a structure with evenly arranged parallel hexagonal structure, which can also be proved by high‐resolution transmission electron microscopy (HRTEM) in **Figure** **4**d,e, which also clearly discloses the layer‐by‐layer stacking growth. The SEM images reveal a film‐like morphology (Figure 4a). Furthermore, the nanosheet Co‐BTB‐LB on a Si substrate was characterized by atomic force microscopy (AFM) in Figure 4b, which shows a flat sheet‐like morphology and a domain size with >10 µm in both height and phase images. Cross‐sectional analysis was conducted to reveal a typical thickness of Co‐BTB‐LB of 88 nm in Figure 4c. A hexagonal structure with a lattice distance of ≈0.25 nm is shown in Figure 4e, which is attributed to the interlayer distance of *π*‐*π* conjugated structure in the nanosheet Co‐BTB‐LB. The interplanar spacing of 0.25 nm is attributed to the CoO(111) crystal plane. The as‐synthesized nanosheet Co‐BTB‐LB and bulk Co‐BTB‐HT from the hydrothermal method were examined by powder X‐ray diffraction (PXRD) to investigate the crystallinity and phase purity. As revealed from PXRD, nanosheet Co‐BTB‐LB and bulk Co‐BTB‐HT displayed one main diffraction peak at the 2*θ* value of 34.15°, which can be attributed to the (111) plane of cubic phase CoO (JCPDS No.42‐1300). The PXRD method was also used to investigate the nanosheet Co‐BTB‐LB after soaking in 1 M KOH for 6 h. As shown in Figure S6 (Supporting Information), the two PXRD patterns of Co‐BTB‐LB before and after soaking in 1 M KOH aqueous solution are basically the same, indicating the stability of Co‐BTB‐LB. A very weak Bragg diffraction peak of the bulk Co‐BTB‐HT counterpart was detected at the same position, reflecting that nanosheet Co‐BTB‐LB exhibited a strong preferential orientation (Figure S6, Supporting Information).^[^ ^30^ ^]^

**Figure 5:**
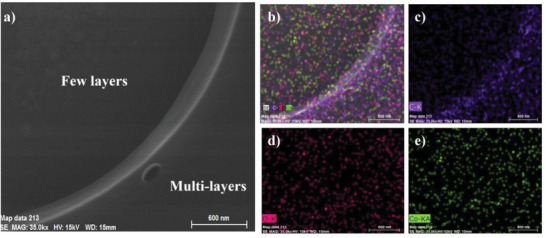
a) The SEM/EDX image of Co‐BTB‐LB on Si substrate. b) The SEM/EDX mapping images of Co‐BTB‐LB for C, O, Co, and c) C d) O e) Co, respectively.

**Figure 6:**
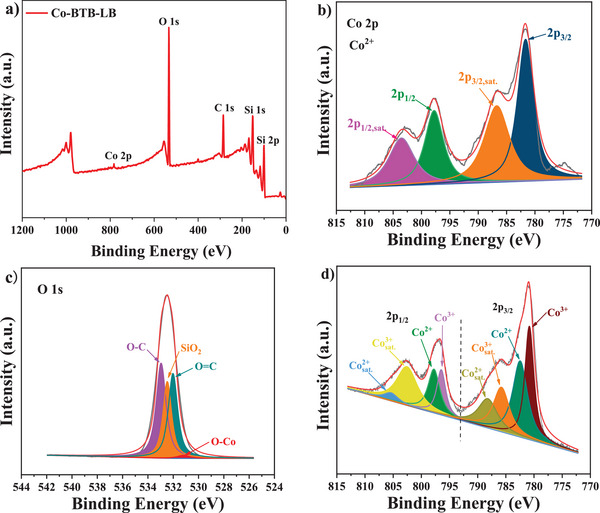
a) The full XPS spectrum of nanosheet Co‐BTB‐LB. The narrow XPS spectra of Co‐BTB‐LB focusing on b) Co 2p, and c) O 1s, respectively. d) The narrow XPS spectra focusing on Co for the product Co‐BTB‐HT.

Besides using various microscopy techniques, energy dispersive X‐ray spectroscopy (EDX) and XPS were also conducted to analyze the chemical elements of nanosheet Co‐BTB‐LB. The SEM/EDX mapping images of Co‐BTB‐LB reveal the homogeneity in the distribution of C, O and Si on Co‐BTB‐LB as proposed (**Figure** **5**). Additionally, XPS was used to further investigate the internal structure and electronic surface state of Co‐BTB‐LB and the product of the reaction by hydrothermal method (**Figure** **6**). As shown in Figure 6a, Co‐BTB‐LB contains C, O, and Co. The valence state of Co ion in Co‐BTB‐LB is determined to be +2 (Figure 6b). This indicates that only Co^2+^ is present in Co‐BTB‐LB. The binding energies of Co 2p_3/2_ and Co 2p_1/2_ of Co‐BTB‐LB are at 781.7 and 797.8 eV, which are attributed to the peaks at Co2p_3/2_ and Co2p_1/2_ of Co^2+^, respectively.^[^ ^31^ ^]^ Notably, two other apparent satellite (sat.) peaks at 786.7 and 803.5 eV are related to the Co^2+^ arising from the chemical reaction due to the coordination of Co^2+^ with carboxylic oxygen from the H_3_BTB ligand.^[^ ^32^ ^]^ As shown in Figure 6c, besides the binding energies of 532.9 and 532.0 eV corresponding to the C—O and C=O bonds from carboxylic group, there is a binding energy peak at 531.2 eV, which indicates that there is Co—O bond in Co‐BTB‐LB and the successful coordination between Co^2+^ and H_3_BTB ligand.^[^ ^33^ ^]^ To investigate the narrow XPS spectra focusing on Co for the product Co‐BTB‐HT by hydrothermal method (Figure 6d), the two peaks located at 782.5 and 797.7 eV suggest the existence of Co^2+^, while the binding energies of 780.8 and 796.5 eV are attributed to Co^3+^.^[^ ^34^ ^]^ It is because at high temperature and high pressure in hydrothermal reaction, the reactants are prone to form clusters, which promote part of the original Co^2+^ ions to be oxidized to Co^3+^ ions.

In order to investigate their adsorption–desorption behavior, the Brunauer‐Emmett‐Teller (BET) tests were conducted for both nanosheet Co‐BTB‐LB and bulk Co‐BTB‐HT (Figure S7, Supporting Information). The specific surface areas of nanosheet Co‐BTB‐LB and bulk Co‐BTB‐HT were calculated to be 95.94 and 14.30 m^2^ g^−1^, respectively. The former one is nearly seven times higher than that of the latter counterpart, which may be attributed to the notion that the mild bottom‐up synthesis method for nanosheet Co‐BTB‐LB is much more favorable to the formation of a superstructure with ordered nanopores and layered structure.

### 2.2 Electrochemical Properties of Nanosheet Co‐BTB‐LB

**Figure 7:**
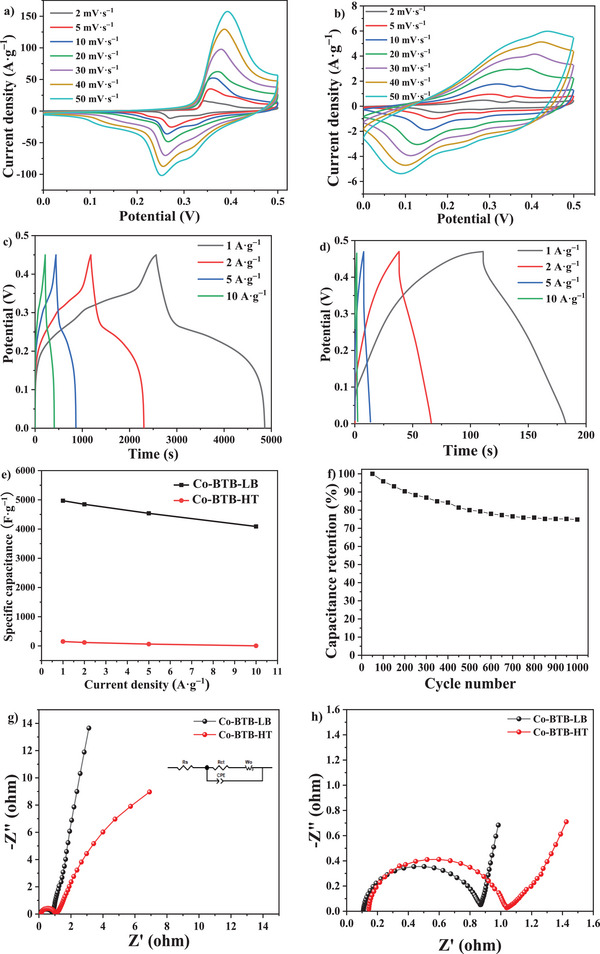
a) CV curves of nanosheet Co‐BTB‐LB at different scan rates from 2 to 50 mV s^−1^. b) CV curves for Co‐BTB‐HT at different scan rates from 2 to 50 mV s^−1^. c) GCD curves of nanosheet Co‐BTB‐LB at different current densities of 1, 2, 5, and 10 A g^−1^. d) GCD curves of Co‐BTB‐HT at different current densities of 1, 2, 5, and 10 A g^−1^. e) Specific capacitance curves of Co‐BTB‐LB and Co‐BTB‐HT at different current densities. f) Cycling stability test of Co‐BTB‐LB. g) Comparison of the EIS Nyquist plots for Co‐BTB‐LB and Co‐BTB‐HT electrodes. h) The high‐frequency region data for (g).

Since the superstructure with ordered nanopores and layered structure can provide much more active sites for the as‐synthesized nanosheet Co‐BTB‐LB, it is expected to be useful in supercapacitor application. To evaluate the electrochemical performance of Co‐BTB‐LB electrode, the electrode with an initial mass loading of 0.15 mg was prepared and the electrochemical measurements were investigated in 1 M KOH electrolyte. The cyclic voltammetry (CV) performance of the Co‐BTB‐LB electrode at different scan rates from 2 to 50 mV s^−1^ is shown in **Figure** **7**a. According to the shapes of the curves, it is obvious that there are a pair of redox peaks which are originated from the reversible redox reaction for Co^2+^/Co^3+^. This suggests the pseudocapacitance behavior of the Co‐BTB‐LB electrode which comes from the faradaic redox reactions of various cobalt oxidation states and the possible cobalt conversion involved in the possible charge and the discharge mechanism of Co‐BTB‐LB is depicted in (1) and (2) as follows (where s and ad in the formula represent the solid state and adsorption state, respectively).^[^ ^35^ ^]^

(1) $$\mathrm{Co}{\left(\mathrm{II}\right)}_{s}+{\mathrm{OH}}^{-}↔\mathrm{Co}\left(\mathrm{II}\right){\left(\mathrm{OH}\right)}_{\mathrm{ad}}+e^{-}$$

(2) $$\mathrm{Co}\left(\mathrm{II}\right){\left(\mathrm{OH}\right)}_{\mathrm{ad}}↔\mathrm{Co}\left(\mathrm{III}\right){\left(\mathrm{OH}\right)}_{\mathrm{ad}}+e^{-}$$

The area of the CV and its current response also increase significantly as the scan rate increases, which indicates that Co‐BTB‐LB and Co‐BTB‐HT are beneficial to the fast and reversible Faradaic reaction (Figure 7a,b). The capacitance is positively correlated with the integral area surrounded by the CV curve. By contrast with Co‐BTB‐HT, nanosheet Co‐BTB‐LB demonstrates a larger CV area, indicating that Co‐BTB‐LB owns a higher capacitance.^[^ ^36^ ^]^

In addition, galvanostatic charge‐discharge (GCD) technique was applied to characterize the specific capacitance of the electrodes. The GCD curves of the nanosheet Co‐BTB‐LB and bulk Co‐BTB‐HT at the current density of 1, 2, 5, and 10 A g^−1^ with the voltage window of 0‐0.45 V are shown in Figure 7c. The shapes of the charge and discharge curves for sharp sloping segments and gentle plateaus are not the standard triangle but a distorted one, which is consistent with the CV results above, and indicates that the origin of capacitance is closely related to a faradaic process. This further reveals the good pseudocapacitance behavior of the electrodes of nanosheet Co‐BTB‐LB.^[^ ^37^ ^]^ The discharge times are 2236, 1090, 408, and 184 s for nanosheet Co‐BTB‐LB under the current density of 1, 2, 5, and 10 A g^−1^, respectively (**Table** **1**). Figure 7d shows the constant current charging and discharging curves of Co‐BTB‐HT under the current density of 1, 2, 5, and 10 A g^−1^ with the voltage window of 0−0.47 V. According to the curves, with the continuous increase of current density from 1 to 10 A g^−1^, the area of the constant current charge and discharge curves is getting smaller and smaller, which conforms to the characteristics of pseudocapacitance. Under the current density of 1, 2, 5, and 10 A g^−1^, the discharge times of Co‐BTB‐HT are 71, 28, 6, and 1 s, respectively, which are much lower than that of nanosheet Co‐BTB‐LB. Figure 7e shows a graph that visually reflects the capacities of Co‐BTB‐LB and Co‐BTB‐HT at different current densities, and the following expression (3) is applied to calculate the electrode specific capacitance in the three‐electrode configuration based on the GCD test data.^[^ ^38^ ^]^

(3) $$C_{s}=\frac{i_{m}\times \Delta t}{\Delta V}$$

where *C*_s_ is the specific capacitance (F g^−1^), Δ*t* is the discharge time (s), *i*_m_ is the current density (A g^−1^) and Δ*V* is the potential window (V). As the current density increases, the specific capacitance of nanosheet Co‐BTB‐LB and sample Co‐BTB‐HT will gradually decrease, because when the current density becomes smaller, there is enough time for OH^−^ to transfer between the electrode surface and the solution, which is of benefit for OH^−^ to embed in or away from the material. In other words, more charge can be transferred and stored, so a higher specific capacitance can be achieved.^[^ ^39^ ^]^ As shown in Table S2 (Supporting Information), the specific capacitance values of 4969.3, 4845.8, 4536.7, and 4088.9 F g^−1^ have been achieved for the nanosheet Co‐BTB‐LB electrode at various current densities of 1, 2, 5, and 10 A g^−1^, respectively. However, at the same current densities as Co‐BTB‐LB, Co‐BTB‐HT electrode exhibits specific capacitance values of 151.9, 118.7, 62.8, and 3.8 F g^−1^ at various current densities of 1, 2, 5, and 10 A g^−1^, which are far lower than that of the nanosheet Co‐BTB‐LB electrode. Table 1 compares the specific capacitance of nanosheet Co‐BTB‐LB with other reported supercapacitors using different synthesis methods. The nanosheet Co‐BTB‐LB has the highest specific capacitance, probably because the specific surface area of Co‐BTB‐LB is much larger than that of Co‐BTB‐HT so that Co‐BTB‐LB can provide a larger number of active sites. Compared with the uniform and continuous pore structure of nanosheet Co‐BTB‐LB, the agglomerated particles are not conducive for OH^−^ to penetrate into Co‐BTB‐HT and react with the active sites, which is harmful to the adsorption and desorption of OH^−^, and makes it impossible for electrons to be transmitted on time. As shown in Figure 7f, a total of 1000 cycles of GCD tests were performed on the sample Co‐BTB‐LB electrode at 10 A g^−1^ in the potential range of 0 to 0.45 V. The specific capacitance of the test sample can be stabilized at ≈75% of the initial value after 750 cycles until 1000 cycles. To further compare the fundamental capacitive behaviors of the two electrode materials for supercapacitors, the electrochemical impedance spectra (EIS) tests were also performed to analyze the resistance, ionic conductivity, charge, and mass transfer for nanosheet Co‐BTB‐LB and sample Co‐BTB‐HT. The EIS Nyquist plot is generally composed of a semicircle in the high frequency region and a straight line in the low frequency region. The semicircle corresponds to the charge transfer resistance (*R*_ct_), the straight line reflects the diffusion resistance of the electrolyte, and a constant phase element (CPE) is used to compensate for the dispersion effect. Electrolyte resistance (*R*_s_) which is generated from the resistance between electrode material and electrolyte reflects the conductive discrepancy of the material. Diffusion can also cause impedance, called Warburg impedance (*W*_o_), which depends on the frequency of the potential disturbance.^[^ ^40^, ^41^ ^]^ As shown in Figure 7g–h, the tiny semicircle (*R*_ct_ = 0.75 Ω) in the high frequency region suggests a fast charge transfer in Co‐BTB‐LB electrode. The inclination angle of the curve is obviously >45° in the low frequency region, showing a more obvious diffusion process in this region. The small electrolyte resistance (*R*_s_ = 0.11 Ω) suggests the low resistance between the electrode material and electrolyte during the electrochemical reaction, suggesting the excellent charge transfer performance for nanosheet Co‐BTB‐LB. This is because the interlayer space of Co‐BTB‐LB and the micro/mesopore formed by the stacking of Co‐BTB‐LB nanosheets is conducive to the migration of OH^−^. Compared to nanosheet Co‐BTB‐LB, the values of *R*_ct_ and *R*_s_ for Co‐BTB‐HT are 0.88 and 0.15 Ω, respectively, which are greater than that of nanosheet Co‐BTB‐LB, indicating a relatively lower charge transfer.

**Table 1:** Comparison of the specific capacitance of supercapacitors with different synthetic methods for previous reports and this work

Metal	Ligand	Synthetic Method	Specific Capacitance	Electrolyte	Reference
Co	H_3_BTB	Bottom‐up method	4969.3 F g^−1^@1A g^−1^	1 M KOH	This work
Co	H_3_BTB	Solvothermal method	152 F g^−1^@1A g^−1^	1 M KOH	This work
Co	H_3_BTC	Grinding followed by oxidation	608.2 F g^−1^@0.25 A g^−1^	1 M KOH	[42]
Co	H_3_BTC	Solvothermal method	958.1 F g^−1^@2A g^−1^	3 M KOH	[43]
Co	H_2_BDC	High temperature stirring	2564 F g^−1^@1 A g^−1^	5 M KOH	[44]
Co	H_2_F_4_BDC hmt	Mixed synthesis	2474 F g^−1^ @1 A g^−1^	1 M KOH	[35]
Co	H_3_TATBA	Solvothermal synthesis	512 F g^−1^@1 A g^−1^	3 M KOH	[45]
Ni	HITP	Hydrothermal method	111 F g^−1^ @0.5 A g^−1^	1 M TEABF4/ACN	[46]
Co	CATB‐6	Hydrothermal method	334 F g^−1^ @1 A g^−1^	3 M KOH	[47]
Ni	H_2_BDC	Hydrothermal method	804 F g^−1^@1 A g^−1^	2 M KOH	[48]
Cu	DBC	Hydrothermal method	479 F g^−1^@0.2 A g^−1^	1 M KCl	[49]
Cu	CAT	Hydrothermal method	463 mF cm^−2^ @1.25 mA cm^−2^	3 M NaCl	[50]
Ni/Co	PTA (H_2_BDC	Facile ultrasonication at room temperature	1202.1 F g^−1^@1 A g^−1^	2 M KOH	[51]
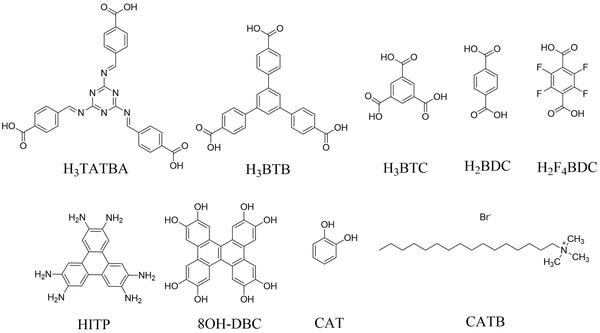					

GCD curves of Co‐BTB‐LB electrodes with mass loadings of 0.70 and 0.98 mg have also been tested at different current densities of 1, 2, 5, and 10 A g^−1^ (Figure S8b,c and Table S2, Supporting Information). According to the calculation using formula (3), the respective specific capacitance values of 4871.58 and 4941.24 F g^−1^ have been achieved for the Co‐BTB‐LB electrodes at the current density of 1 A g^−1^, which are similar to that of the Co‐BTB‐LB electrode with a mass loading of 0.15 mg (4969.3 F g^−1^) under the same condition, indicating that the relatively low mass loading at 0.15 mg still provides accurate enough specific capacitance of the Co‐BTB‐LB electrode. Furthermore, in order to eliminate the influence of Ni foam on the specific capacitance, a contrast test of blank Ni foam has been made. Under the same test conditions, the discharger time for blank Ni foam is 16.4 s at 1 A g^−1^ and the specific capacitance value is only 39 F g^−1^ (Figure S8d, Supporting Information). Compared with nanosheet Co‐BTB‐LB, the specific capacitances for Ni foam are too small, so the specific capacitances generated by  Ni foam can be negligible.

### 2.3 Electrochemical Performance of Co‐BTB‐LB//AC (active carbon) Based Asymmetric Supercapacitor (ASC)

**Figure 8:**
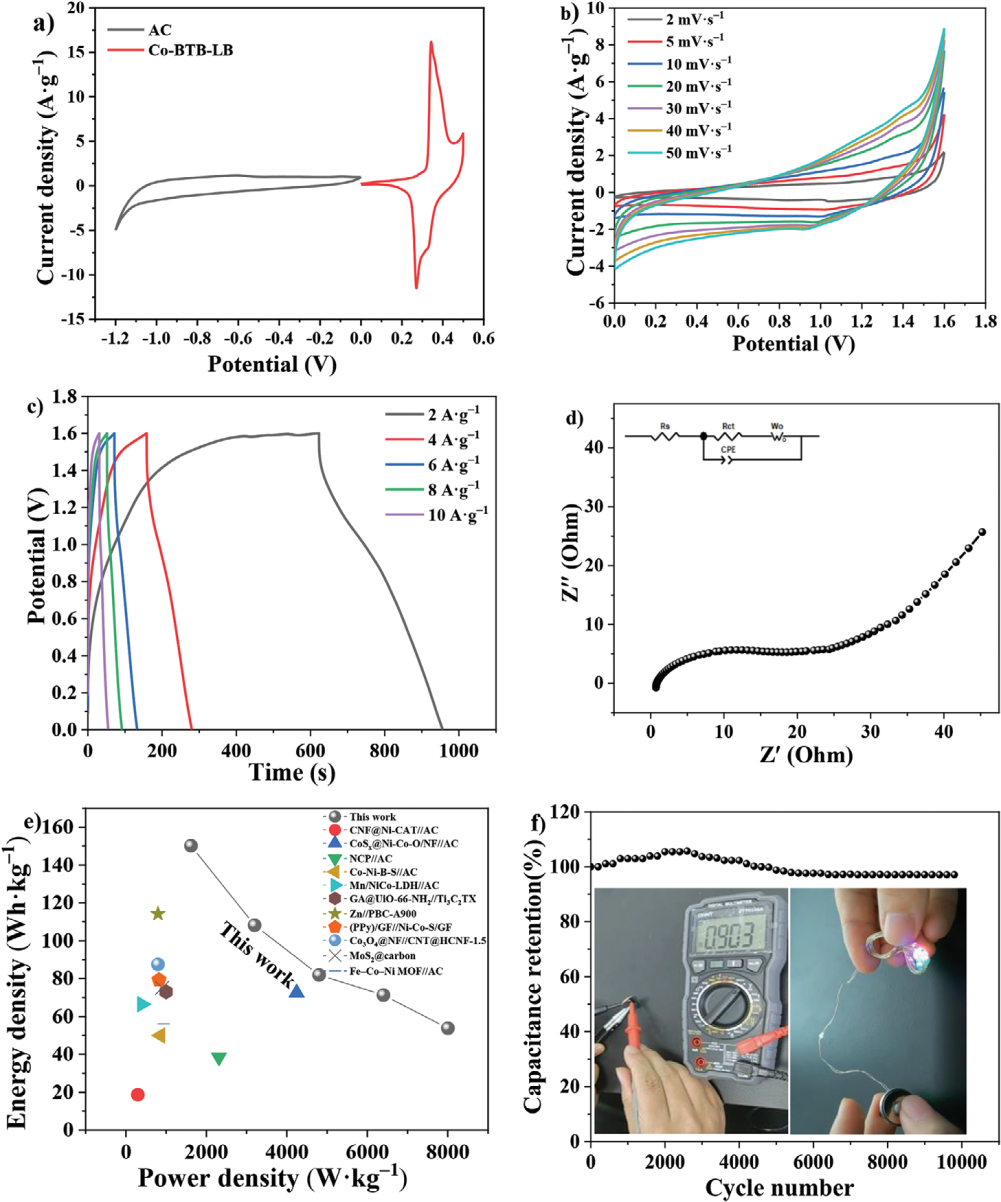
a) Comparison of CV curves between Co‐BTB‐LB and AC electrodes at 2 mV s^−1^. Electrochemical performances of Co‐BTB‐LB//AC based ASC device. b) CV curves with different potentials at different scan rates from 2 to 50 mV s^−1^. c) GCD curves with different current densities from 2 to 10 A g^−1^. d) EIS Nyquist plots. e) Comparison of energy density and power density with other reported ASC materials. f) Cycling stability test at the current density of 10 A g^−1^. Inset: Schematic for lighting up the light‐emitting diodes by Co‐BTB‐LB//AC based ASC device.

In order to evaluate the application prospect of the electrode, ASC device was prepared, which was composed of the Co‐BTB‐LB electrode, AC, and 6 M KOH aqueous solution, and was used as the positive electrode, negative electrode, and electrolyte, respectively. According to equations (4) and (5), the energy and power density of the Co‐BTB‐LB electrode could be obtained as follows:^[^ ^40^, ^41^ ^]^

(4) $$E=\frac{1}{2}\times \frac{C_{s}\times V^{2}}{3.6}$$

(5) $$P=\frac{3600\;E}{\Delta t}$$

where *E* is the energy density (Wh kg^−1^), *P* is the power density (W kg^−1^), *C_s_* is the specific capacitance (F g^−1^), *V* is the voltage and Δ*t* is the discharge time (s). **Figure** **8**a shows the classical CV curves of AC and Co‐BTB‐LB electrodes at 2.0 mV s^−1^. Based on the CV curves of Co‐BTB‐LB and AC in the three‐electrode system and the ASC device, the ASC device can easily reach a voltage of 1.8 V (Figure S9, Supporting Information). Considering the balance among different electrochemical properties of the device, we selected a potential window of 0–1.6 V for the measurements. The CV curves of the as‐prepared ASC device in a series of sweep rates of 0–1.6 V are depicted in Figure 8b. It is clear to see that there are redox peaks in the quasi‐rectangular CV curves, which testify the effective integration of pseudocapacitance and double‐layer capacitance.^[^ ^41^, ^52^ ^]^ Furthermore, the GCD curves were recorded and the specific capacitance of the Co‐BTB‐LB//AC based ASC were calculated to be 417.3, 304.5, 230.3, 200.5, and 151.3 F g^−1^ at different discharge current densities of 2, 4, 6, 8, and 10 A g^−1^ respectively. (Figure 8c, **Table** **2**). Meanwhile, the EIS profile for the as‐prepared ASC device exhibits a very low *R*_s_ of 0.73 Ω (Figure 8d), which verifies that low internal resistance and inherent porosity are favorable for the ions to approach the active center.^[^ ^50^ ^]^ From Figure 8e and Table 2, the as‐prepared ASC device exhibits a maximum energy density of 150.2 Wh kg^−1^ at a power density of 1619.2 W kg^−1^ and 53.8 Wh kg^−1^ at 8003.3 Wkg^−1^, demonstrating that the ASC device keeps both high power density and energy density, which is superior to most other reported ASC materials, such as Co‐Ni‐B‐S//AC,^[^ ^18^ ^]^ MoS_2_//carbon,^[^ ^19^ ^]^ CoS_x_@Ni‐Co‐O/NF//AC,^[^ ^34^ ^]^ NCP//AC,^[^ ^40^ ^]^ CNF@Ni‐CAT//AC,^[^ ^53^ ^]^ Mn/NiCo‐LDH//AC,^[^ ^54^ ^]^ GA@UiO‐66‐NH_2_//Ti_3_C_2_T_X_,^[^ ^55^ ^]^ Zn//PBC A900,^[^ ^56^ ^]^ (PPy)/GF//Ni‐Co‐S/GF,^[^ ^57^ ^]^ Co_3_O_4_@NF//CNT@HCNF‐1.5,^[^ ^58^ ^]^ and Fe‐Co‐Ni MOF//AC^[^ ^59^ ^]^ (Figure 8e). Furthermore, the as‐prepared ASC device reveals a good cycling stability with a capacitance retention of 97.1% after 10 000 cycles performed at a current density of 10 A g^−1^ (Figure 8f). Light‐emitting diodes were successfully lighted up by connecting two as‐prepared ASC devices in series (inset of Figure 8f), demonstrating the potential applications of nanosheet Co‐BTB‐LB in energy‐storage devices.

**Table 2:** Specific capacitance, energy density and power density of Co‐BTB‐LB//AC based ASC at different current densities

Current density [A g^−1^]	Specific capacitance [F g^−1^]	Energy density [Wh kg^−1^]	Power density [W kg^−1^]
2	417.3	150.2	1619.9
4	304.5	108.2	3200.7
6	230.3	81.9	4801.9
8	200.5	71.3	6401.0
10	151.3	53.8	8003.3

## 3 Conclusion

A 2D MOF nanosheet Co‐BTB‐LB has been successfully synthesized by a facile liquid–liquid interface‐assisted method. The as‐prepared nanosheet Co‐BTB‐LB has good structural stability and large size domains, which have been identified by SEM/EDX and XPS. The sheet‐like structure of nanosheet Co‐BTB‐LB has been verified by SEM, HRTEM, and AFM, revealing a flat morphology and a domain size with >10 µm in both height and phase images, and the cross‐sectional analysis was conducted to a typical thickness of 88 nm. The nanosheet Co‐BTB‐LB can show a high utilization of the redox‐active sites, good electrochemical stability and energy storage properties, relative to the corresponding powder sample Co‐BTB‐HT by hydrothermal method, which is indicative of the superiority of the MOF nanosheet prepared by the liquid–liquid interface‐assisted approach. Co‐BTB‐LB electrode exhibits good electrochemical properties of high capacity of 4969.3 F g^−1^ at the current density of 1 A g^−1^ and good cycling stability with 75% capacity retention after 1000 cycles. The ASC device with Co‐BTB‐LB as the positive electrode shows a maximum energy density of 150.2 Wh kg^−1^ at a power density of 1619.2 W kg^−1^ and good cycling stability with a capacitance retention of 98% after 5000 cycles performed at a current density of 10 A g^−1^, demonstrating the potential applications of nanosheet Co‐BTB‐LB in energy‐storage devices. To our knowledge, this is a state‐of‐the‐art performance reported so far for electroactive bottom‐up metal‐complex nanosheet applied to supercapacitor, which will clearly lead to a significant expansion of the applicability of this type of 2D nanomaterials as functional nanomaterials.

## Conflict of Interest

The authors declare no conflict of interest.

## Supporting information

Supporting Information

## Data Availability

The data that support the findings of this study are available from the corresponding author upon reasonable request.
