# Supplementary material for: A Cobalt‐Based Metal‐Organic Framework Nanosheet as the Electrode for High‐Performance Asymmetric Supercapacitor
Source: Adv Sci (Weinh). 2023 Apr 23;10(18):2207545. doi: 10.1002/advs.202207545 (PMC10288240; doi:10.1002/advs.202207545)
Supplement: Supplementary file 1 — Supporting Information [file ADVS-10-2207545-s001.pdf]

ADVANCED SCIENCE
Open Access

Supporting Information

for *Adv. Sci.*, DOI 10.1002/advs.202207545

A Cobalt-Based Metal-Organic Framework Nanosheet as the Electrode for High-Performance Asymmetric Supercapacitor

Qian Liu, Zengqi Guo, Cong Wang, Su Guo, Zhiwei Xu, Chenguang Hu, Yujing Liu, Yalei Wang, Jun He and Wai-Yeung Wong**

ADVANCED SCIENCE
Open Access

Supporting Information

for *Adv. Sci.*, DOI 10.1002/advs.202207545

A Cobalt-Based Metal-Organic Framework Nanosheet as the Electrode for High-Performance Asymmetric Supercapacitor

Qian Liu, Zengqi Guo, Cong Wang, Su Guo, Zhiwei Xu, Chenguang Hu, Yujing Liu, Yalei Wang, Jun He and Wai-Yeung Wong**

Supporting Information

A Cobalt-Based Metal-Organic Framework Nanosheet as the Electrode for High-Performance Asymmetric Supercapacitor

Qian Liu, Zengqi Guo, Cong Wang, Su Guo, Zhiwei Xu, Chenguang Hu, Yujing Liu, Yalei Wang, Jun He, Wai-Yeung Wong**

1. Experimental section

1.1. Chemicals and reagents

All chemical reagents were analytically pure and were used directly without purification or treatment. Cobalt(II) nitrate hexahydrate ($Co(NO_3)_2{\cdot}6H_2O$, > 99.0%), ethanol (CH_3CH_2OH, AR, 95%), dichloromethane (CH_2Cl_2, > 99.9%), *N,N*-dimethylformamide (DMF, AR, ≥ 99.9%), isopropanol (HPLC, 99.8%) and poly(tetrafluoroethylene) (PTFE, RG, 60-62 wt.% dispersion) were obtained from Shanghai Titan Scientific Co., Ltd, 1,3,5-tris(4-carboxyphenyl)benzene (H_3BTB, ≥ 97.0%), potassium hydroxide (KOH, 95%) and acetylene black (≥ 99.9%) were obtained from Shanghai Aladdin Biochemical Technology Co., Ltd. The water used for the preparation of the solution was deionized (D.I.) water.

1.2. Apparatus for characterization

Scanning electron microscope and energy dispersive X-ray spectroscopy (SEM and EDX, Hitachi S-4800) were carried out to observe the morphologies and elemental distributions of nanosheet **Co-BTB-LB** at 1.0–2.0 kV and 10–20 kV, respectively. Transmission electron microscopy and high resolution transmission electron microscopy (TEM and HRTEM, ThermoFischer FEI tecnai f20) were carried out to observe the morphologies of **Co-BTB-LB** with the accelerating voltage of 200 kV. Atomic force microscopy (AFM, Shimadzu SPM-9700) was carried out to observe the thickness of **Co-BTB-LB**. At the same time, X-ray photoelectron spectra (XPS) were recorded under 30 eV Al-Kα radiation using ThermoFischer Escalab Xi^+. ^1H NMR spectra were recorded on a Bruker Model AV-500 NMR spectrometer. Chemical shifts (δ) were reported in ppm. The positive-ion electrospray liquid chromatography (LC) mass spectra were recorded in alcohol matrices on the Shimadzu LCMS-8030 mass spectrometer. Infrared spectra (IR) were recorded on the Shimadzu IR Prestige-21 FT-IR spectrometer using KBr pellets for solid state spectroscopy. PXRD was performed on a Bruker D8 Advance X-ray diffractometer, with Cu Kα1 (45 kV, 100 mA).

The N_2 adsorption-desorption measurements were conducted with an ASAP2460 Surface Area and Porosity Analyzer at 150℃.

1.3. Fabrication and characterization of electrodes

Before the preparation of the electrodes, Ni foam (the size of the electro-deposition is 2 cm × 1.5 cm) was soaked in acetone, D.I. water and 0.1 mol/L HCl solution and placed under an ultrasonic cleaner for 10, 5 and 10 min, respectively. Then the Ni foam was dried for 3 h at 60 °C under vacuum after being cleaned repeatedly using D.I. water and ethanol three times, respectively.

All electrochemical properties such as such as cyclic voltammetry (CV), galvanostatic charge-discharge (GCD), electrochemical impedance spectroscopy (EIS) and long-term stability measurements were performed in a three-electrode system containing 1.0 M KOH aqueous solution as the electrolyte on a CHI 660E electrochemical workstation (CH Instruments, Chenhua. Co., Shanghai). Where Ni foam modified with the as-prepared nanosheet **Co-BTB-LB** or slurry containing **Co-BTB-HT** was used as the working electrode, a saturated calomel (SCE) electrode and a platinum sheet were applied as the reference electrode and the counter electrode, respectively. CV was measured within the potential window of 0 to 0.5 V at the scan rates of 2–50 $mV \cdot s^{-1}$. GCD curves were conducted in the potential range of 0–0.5 V at different constant current density within 1–10 $A \cdot g^{-1}$. The cycle life tests were carried out by GCD measurements with a constant current density of 10 $A \cdot g^{-1}$ for 1000 cycles. EIS measurements were performed in a frequency range of 10^{-2}–10^5 Hz at an open circuit potential with an ac perturbation of 5 mV.

The working electrode was prepared for **Co-BTB-HT** according to the literature methods as follows: the acquired **Co-BTB-HT** materials, acetylene black and PTFE with a mass ratio of 8:1:1 were mixed with isopropanol to form a homogeneous slurry. Afterwards, the slurry was coated on an as-prepared Ni foam electrode. Finally, the electrode was allowed to dry at room temperature for 3 h. All the electrochemical studies were carried out under ambient conditions. The mass loadings of **Co-BTB-LB** on each substrate were 0.15, 0.70 and 0.98 mg, and that of **Co-BTB-HT** was 10.29 mg, which were calculated by the mass difference before and after the coating. All materials were weighed in a millionth high-precision analytical balance (Model: RADWAG XA 4Y.M.A).

1.4. Fabrication and characterization of Co-BTB-LB//AC based asymmetric supercapacitor (ASC)

For evaluating the application prospect of the electrode, asymmetric supercapacitor (ASC) should be prepared. An asymmetric supercapacitor consists of the **Co-BTB-LB** electrode, AC, and 6 M KOH aqueous solution, which was employed as the positive electrode, negative electrode, and electrolyte, respectively. The negative electrode was prepared for AC according to the literature method as follows: the acquired AC, acetylene black and PTFE with a mass ratio of 8:1:1 were mixed in isopropanol to form a homogeneous slurry. Afterwards, the slurry was coated on an as-prepared Ni foam electrode. Finally, the electrode was allowed to dry at room temperature for 3 h. The button battery shells and water-based diaphragms of the devices were purchased from the Self-Reliance Battery Sales Department. The model of the shell is LIR2016, which is made of 304 stainless steel, and the diameter and thickness are 20 mm and 1.6 mm. The positive electrode is made of a foam nickel slice with 12 mm in diameter, which is loaded with nanosheet **Co-BTB-LB** and the area loading is about 0.12 $mg \cdot cm^{-2}$. The negative electrode is made of a foam nickel slice 16 mm in diameter, which is loaded with AC, and the area loading is about 0.75 $mg \cdot cm^{-2}$. The water-based diaphragm is made of PP/PE, EVOH and special nylon fiber with a thickness of 150 μm, which is cut into a round piece with a diameter of 20 mm for the preparation of ASC device. 2~3 drops 6 M KOH (about 0.3 mL) was used for the electrolyte. The mass loading of AC was obtained by the principle of charge balance. The mass loading of active materials used for positive and negative electrodes must abide the charge balance relationship ($Q+ = Q-$) based on the following equation:

$$\frac{M^{+}}{M^{-}} = \frac{C^{-} \times \Delta V^{-}}{C^{+} \times \Delta V^{+}}$$

The values of specific capacitance and potential window are based on the GCD curves of AC electrode in three-electrode system at the current density of 5 $A \cdot g^{-1}$. Hence:

m(**Co-BTB-LB**)/m(AC) = (293.5 $F \cdot g^{-1} \times$ 1.0 V) : (4536.7 $F \cdot g^{-1} \times$ 0.45 V) ≈ 0.14 : 1

2. Synthesis and characterization of M1

3,5-Diisopropylbenzoic acid (206.3 mg, 1 mmol) and $Co(NO_3)_2·6H_2O$ (145.5 mg, 0.5 mmol) were dissolved in 20 mL of CH_2Cl_2 and 10 mL of D.I. water, respectively. Then a mixture of the two solutions was stirred at room temperature overnight, and the reaction mixture was washed by D.I. water several times. After that, CH_2Cl_2 was evaporated off. The residue was purified by silica gel column using *n*-hexane/ethyl acetate (6:1, v/v) as eluent to provide **M1** as a light green solid (146.5 mg, 52 %). ^{1}H NMR (500 MHz, DMSO-d$_6$): δ = 12.84 (s, 2H, COOH), 7.80 (s, 4H, Ar), 7.67 (s, 2H, Ar), 1.32 (s, 36H, CH_3); IR (KBr) (cm^{-1}): v = 3429 (s, br, v_{OH} (H_2O)), 798–783(s, v_{OH} (H_2O)), 1683 (s, $v_{C=O}$), 1595 (w, $v_{C=C}$), 1475 (w, $v_{C\text{-}O}$), 596 (s, $v_{Co\text{-}O}$); LC-MS (*m/z*): 282.15 $[M]^{2+}$, 274.30 $[M\text{-}H_2O]^{2+}$.

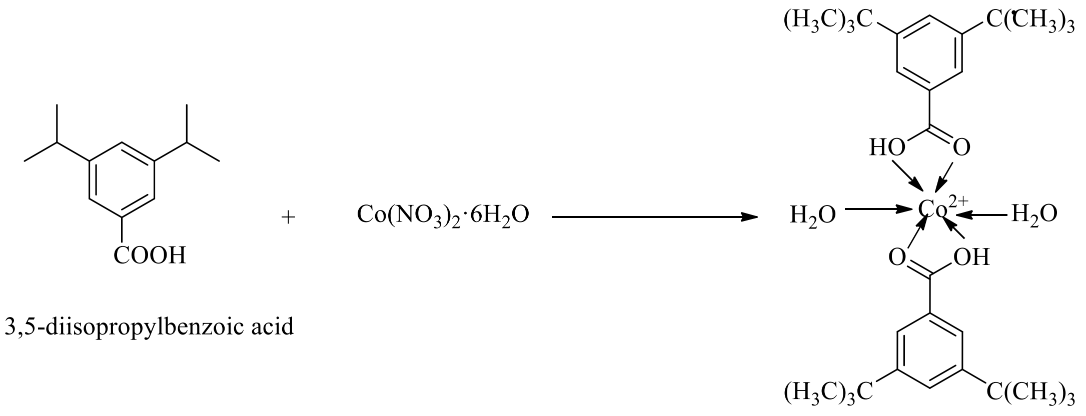

M1

Scheme S1 Synthetic pathway to the molecular model compound **M1**.

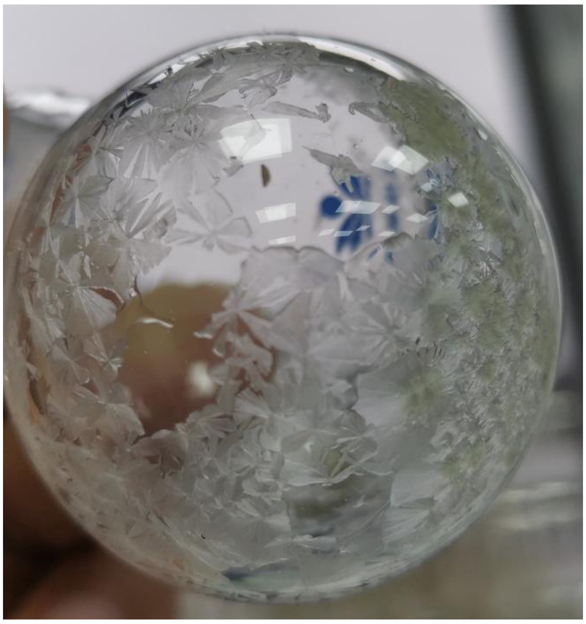

Figure S1 Photograph of compound **M1**.

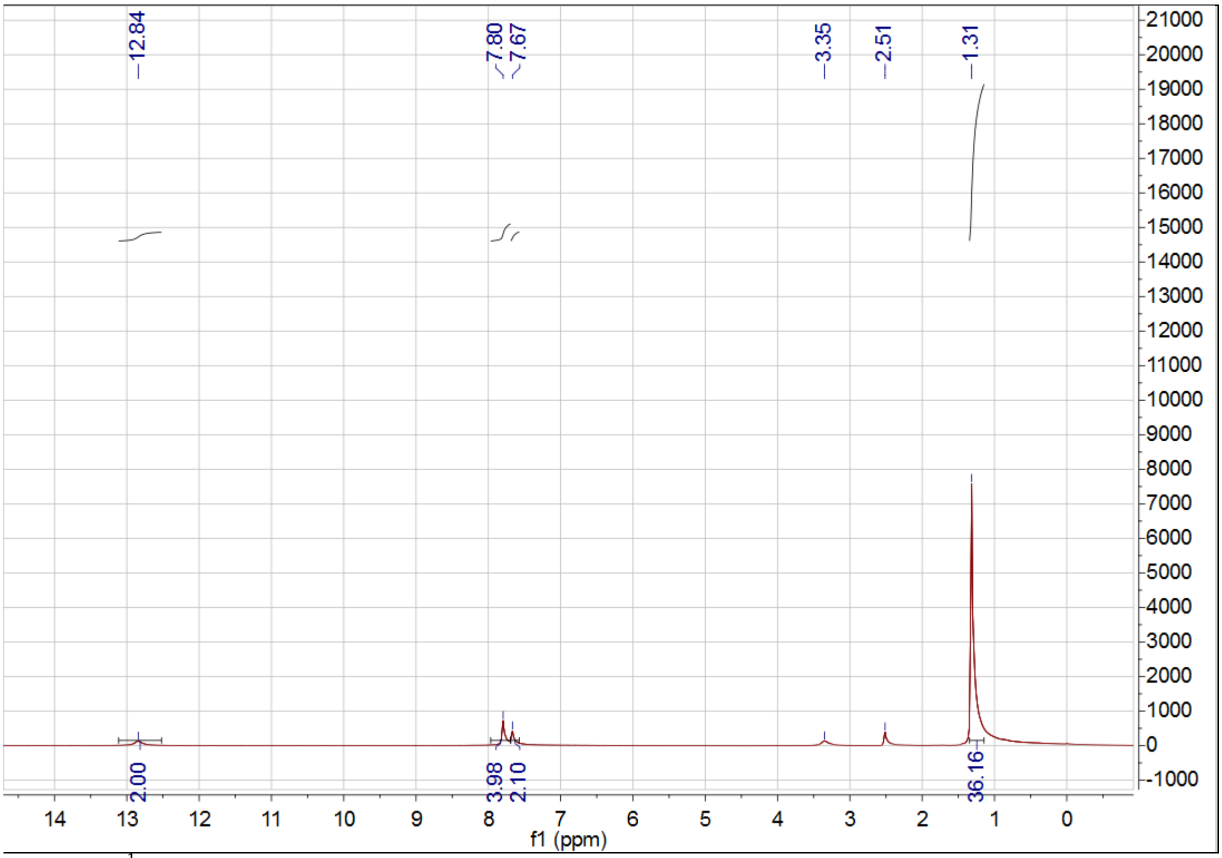

Figure S2 ^{1}H NMR spectrum of **M1** in DMSO-d$_6$.

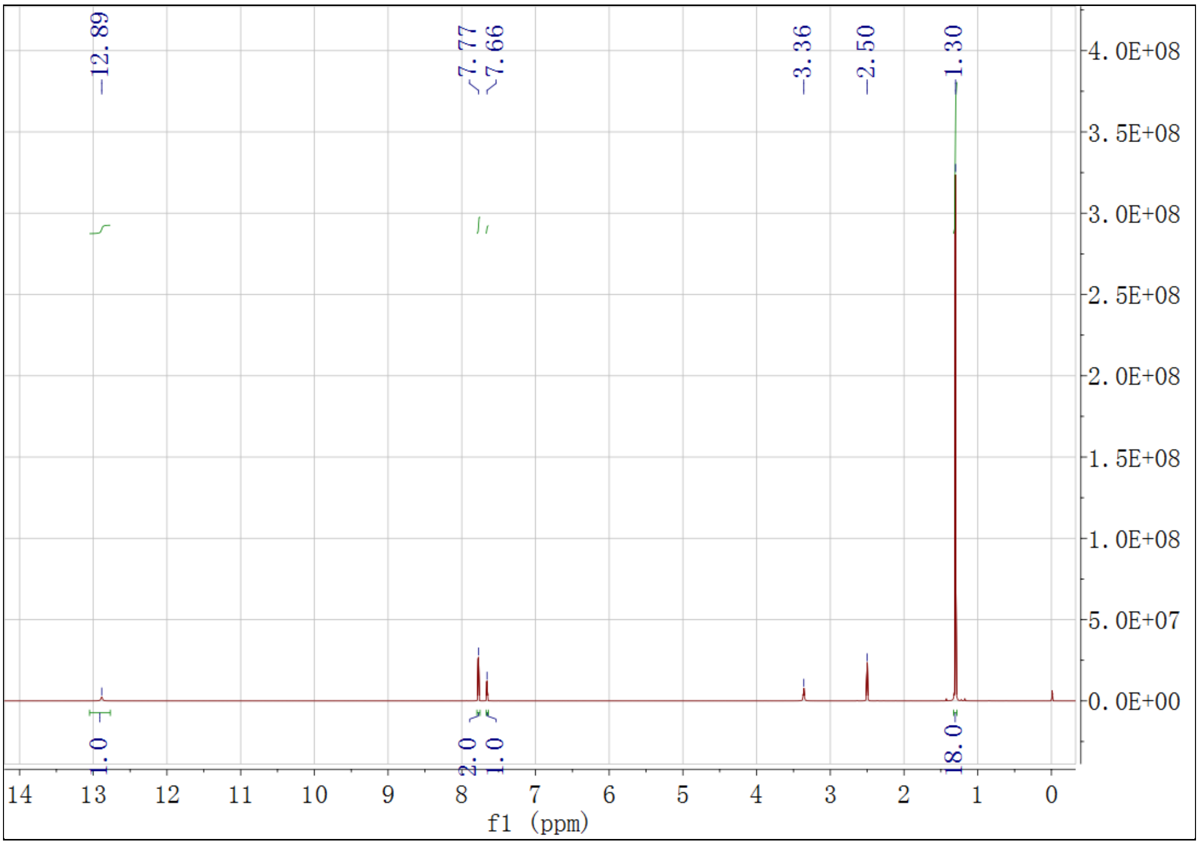

Figure S3 ^{1}H NMR spectrum of ligand 3,5-diisopropylbenzoic acid in DMSO-d_6.

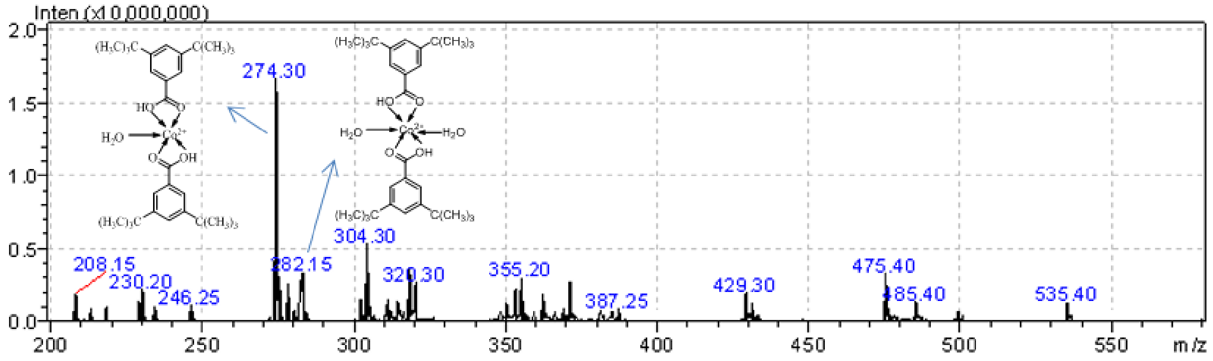

Figure S4 The mass spectrum of compound **M1**.

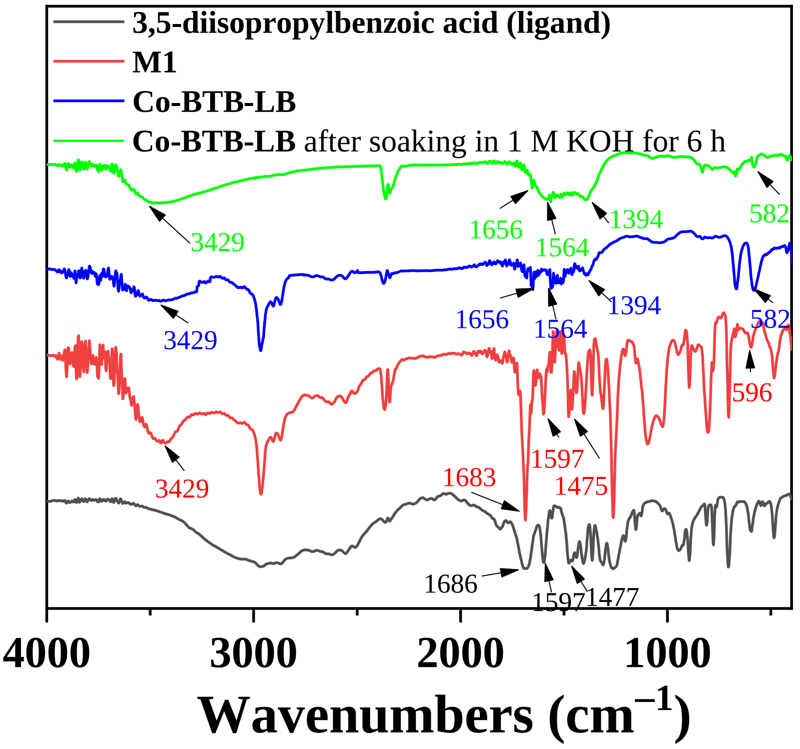

Figure S5 The FT-IR spectra of ligand 3,5-diisopropylbenzoic acid, **M1**, nanosheet **Co-BTB-LB** and **Co-BTB-LB** after soaking in 1 M KOH aqueous solution for 6 h. The two FT-IR spectra of **Co-BTB-LB** before and after soaking 1 M KOH aqueous solution are basically the same, only Ar-COOH vibrational band is missing because of the presence of OH^{-}, indicating the stability of **Co-BTB-LB**.

Table S1 FT-IR spectra of 3,5-diisopropyl-benzoic acid, **M1**, **Co-BTB-LB** and **Co-BTB-LB** after soaking in 1 M KOH aqueous solution for 6 h.

Compound	v_{OH} (H_2O)	$v_{Ar\text{-}COOH}$	$v_{C=O}$	$v_{C=C}$	$v_{C\text{-}O}$	$v_{Co\text{-}O}$
3,5-diisopropyl-benzoic acid	–	3034-2792(s, br), 2659-2504(s, br)	1686(s)	1597(s)	1477(s)	–
**M1**	3429(s, br), 798–783(s)	3034-2792(w), 2659-2504(w, br)	1683(s)	1595(w)	1475(w)	596(s)
**Co-BTB-LB**	3429(s, br), 798–783(s)	3034-2792(w), 2659-2504(w, br)	1656(w)	1564(w)	1394(w)	582(s)
**Co-BTB-LB** (after soaking in 1 M KOH for 6 h)	3429(s, br), 798–783(s)	–	1656(w)	1564(w)	1394(w)	582(w)

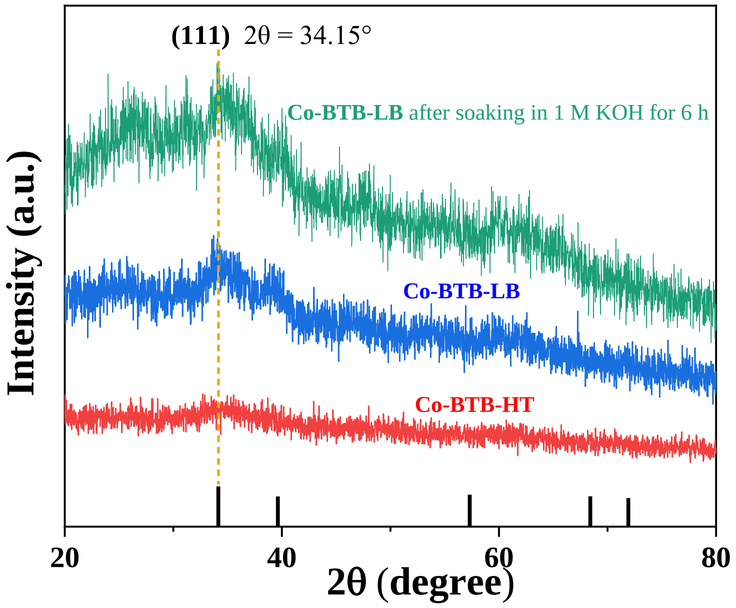

Figure S6 The PXRD patterns of the as-synthesized nanosheet **Co-BTB-LB**, **Co-BTB-LB** after soaking in 1 M KOH for 6 h and bulk **Co-BTB-HT**, respectively. The two PXRD patterns of **Co-BTB-LB** before and after soaking 1 M KOH aqueous solution are basically the same, indicating the stability of **Co-BTB-LB**.

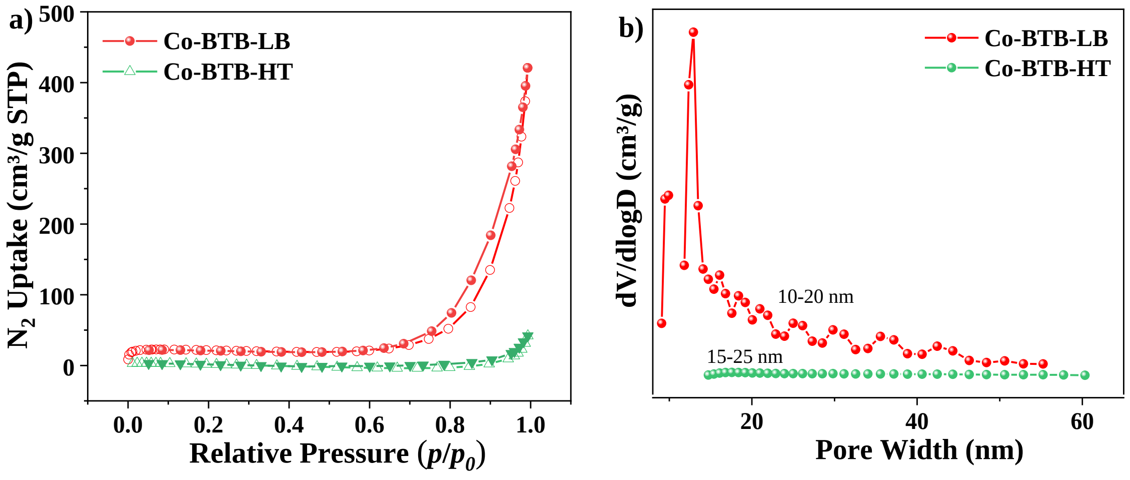

Figure S7 (a) N_2 adsorption-desorption isotherms of nanosheet **Co-BTB-LB** and bulk **Co-BTB-HT** prepared by hydrothermal method. (b) Pore-size distribution plots of nanosheet **Co-BTB-LB** and bulk **Co-BTB-HT**.

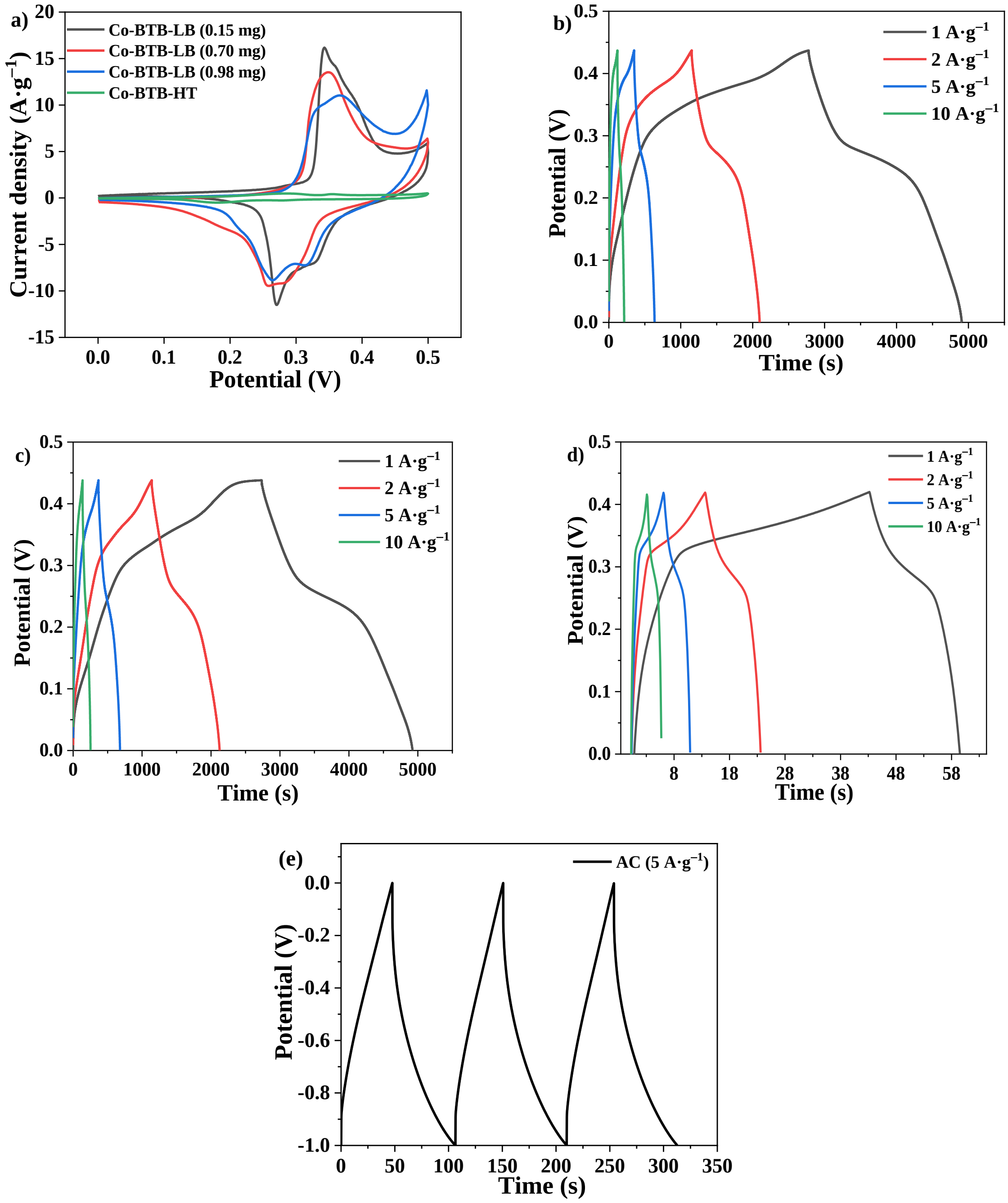

Figure S8 (a) CV curves of nanosheet **Co-BTB-LB** with mass loadings of 0.15, 0.70 and 0.98 mg and bulk **Co-BTB-HT** at different scan rate of 2 mV·s^{-1}. GCD curves of nanosheet **Co-BTB-LB** with mass loadings of (b) 0.70 and (c) 0.98 mg and (d) blank Ni foam at different current densities of 1, 2, 5 and 10 A·g^{-1}.(e) GCD curves of AC electrode in three-electrode system at the current density of 5 A·g^{-1}.

Table S2. Discharge times and specific capacitance for nanosheet **Co-BTB-LB** with different mass loadings of 0.15, 0.70 and 0.98 mg and bulk **Co-BTB-HT** by hydrothermal method with current densities of 1, 2, 5 and 10 A·g^{-1}.

Current Density (A g^{-1})	Discharge Time (s)				Specific Capacitance (F·g^{-1})			
	**Co-BTB-LB**			**Co-BTB-HT**	**Co-BTB-LB**			**Co-BTB-HT**
	0.15 mg	0.70 mg	0.98 mg		0.15 mg	0.70 mg	0.98 mg	
1	2236	2143	2174	71	4969.3	4871.6	4941.2	151.9
2	1090	944	983	28	4845.8	4293.2	4468.2	118.7
5	408	284	312	6	4536.7	3229.5	3550.0	62.8
10	184	93	115	1	4088.9	2131.8	2631.8	3.8

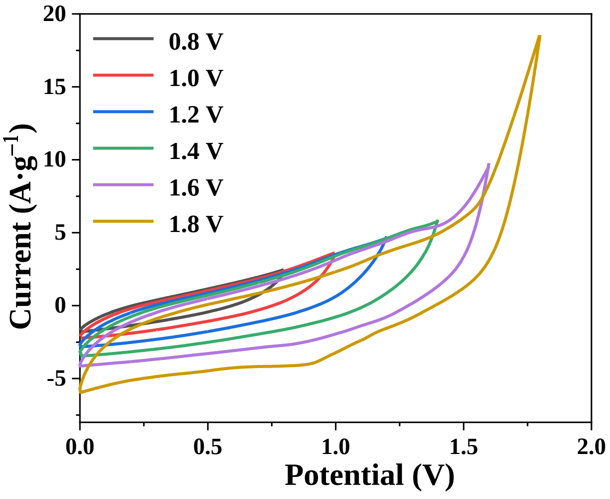

Figure S9 CV curves of the as-prepared ASC device tested with different potential windows.

Reference

[1] Y. C. Yao, Z. Q. Xiao, P. Liu, S. J. Zhang, Y. Niu, H. L. Wu, S. Y. Liu, W. X. Tu, Q. Luo, M. A. Z. G. Sial, S. Z. Zeng, Q. Zhang, J. Z. Zou, X. R. Zeng, W. J. Zhang, *Carbon* **2019**, *155*, 674-685.
